# My History in Juntendo University

**DOI:** 10.14789/jmj.JMJ23-0028-R

**Published:** 2023-11-16

**Authors:** YASUYUKI OKUMA

**Affiliations:** 1 Department of Neurology, Juntendo University Shizuoka Hospital, Shizuoka, Japan; 1 Department of Neurology, Juntendo University Shizuoka Hospital, Shizuoka, Japan; 2 Faculty of Health Science and Nursing, Juntendo University, Tokyo, Japan; 2 Faculty of Health Science and Nursing, Juntendo University, Tokyo, Japan

**Keywords:** neurologist, neurophysiology, Parkinson’s disease, education, faculty development

## Abstract

With my retirement as a professor, I would like to review my 47-year history of studying and working at Juntendo University. I was admitted to Juntendo University School of Medicine in 1976, and after graduation I joined the Department of Neurology in 1982, where Professor Hirotaro Narabayashi was the founding chairman. I became particularly interested in movement disorders and neurophysiology. The second chairman, Professor Yoshikuni Mizuno, established an American-style neurology training system. From 1992 to 1994, I studied electrophysiology at the University of Calgary in Canada, and my family and I enjoyed life in Canada very much. In 2000, I moved to Juntendo Izu-Nagaoka Hospital, now renamed Juntendo Shizuoka Hospital. I instructed young neurologists to write case reports in English. Owing to this achievement, the third chairman, Professor Nobutaka Hattori, recommended me to be a recipient of Alumni Scientific Award and to become a professor of neurology in 2009. I also became an executive officer of the Asian and Oceanian Section of the International Parkinson and Movement Disorders Society from 2015 to 2019. In 2017, I was appointed as the dean of the Faculty of Health Science and Nursing. I devoted myself to improving the nursing education and then I received the Best Professor Award twice. The level of the faculty improved, so that all the students were able to pass the National Nursing Examination consistently. In conclusion, I thank all my colleagues, faculty members, and family for letting me have valuable experiences and memories in Juntendo University.

## Introduction

About 47 years have passed since I was admitted to Juntendo University School of Medicine in 1976. After graduation, I started my career as a neurologist in Juntendo University. Here, I would like to review my history as an undergraduate medical student, a neurologist, and as the dean of the Faculty of Health Science and Nursing until my retirement in March 2023.

## As a medical student

From 1976 to 1982, I spent my student days both studying medicine and playing baseball in the medical school. I enjoyed living in the dormitory, where students of medicine and physical education lived in the same room, in the first year of liberal education. We were very happy because our professors and other faculty members were all excellent and the staff members were so kind. As for baseball, when I was in my 6th year, our baseball team won the gold medal in the Eastern Medical Students Games for the first time. The baseball practice was very hard for me because I had no experience playing baseball competitively. However, I was lucky that the curriculum for medical education was not so tight then.

## My early years as a neurologist

**Figure 1:**
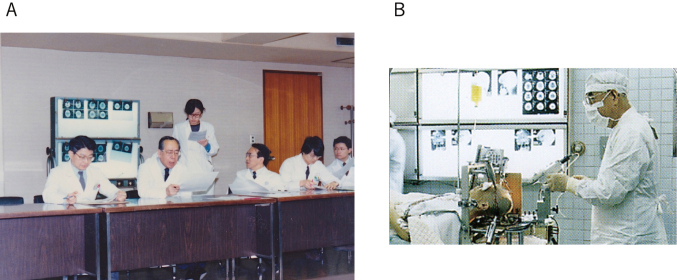
A. Case conference in the Department of Neurology in 1986 From left to right, Associate Professor Takeshi Sato, Professor Hirotaro Narabayashi, Lecturer Hisamasa Imai, Dr. Akito Hayashi, and me B. Professor Narabayashi performing stereotaxic brain surgery at his private clinic

**Figure 2:**
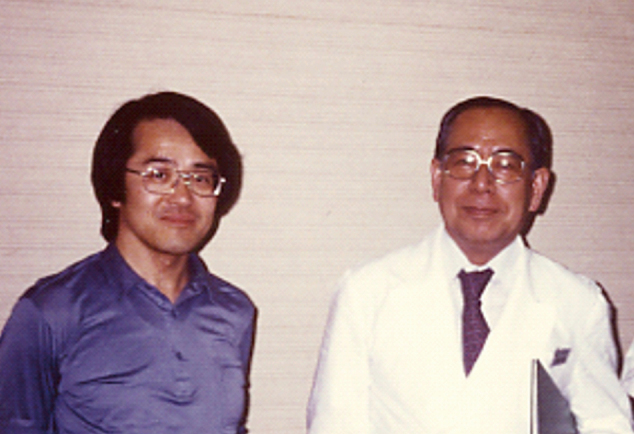
Professor Reisaku Tanaka (left) and Professor Hirotaro Narabayashi (right)

I started my neurology residency at the Department of Neurology, Juntendo University in 1982, where the late Professor Hirotaro Narabayashi was the founding chairman of the department. As he was an expert on Parkinson’s disease, I became interested in movement disorders such as Parkinson’s disease. Figure 1-A shows a picture of a case conference held in 1986. Professor Narabayashi also ran his private clinic for stereotaxic brain surgery, and once a week, I saw patients there before and after operation for 13 years (Figure 1-B). I joined the neurophysiology study group, which consisted of Drs. Masanori Nagaoka, Yasoichi Nakajima, Yasuhiro Kagamihara, and Akito Hayashi, and we used electrophysiological techniques such as the Hoffmann reflex (H-reflex) technique to study motor control. The mentor of this group was the late Professor Reisaku Tanaka (Figure 2), who was working at the Tokyo Metropolitan Institute for Neuroscience. He emphasized eliminating ambiguity and recommended us to read “Scientists must write”^1)^, which immensely influenced me. Since then, I wrote down all my ideas for planning or conducting experimental research.

**Figure 3:**
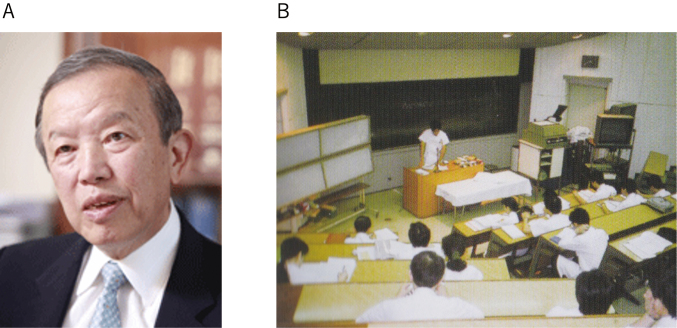
A. Professor Yoshikuni Mizuno B. Conference held every morning

In 1989, Professor Yoshikuni Mizuno became the second chairman of the Department of Neurology (Figure 3-A). Professor Mizuno completed his residency in Chicago, so we were able to learn American-style neurology. Figure 3-B shows a picture of a conference held every morning. Young physicians worked till midnight to prepare for the next day’s morning conference. Two years later, I was appointed as a chief physician of the neurology ward, and the Chief Resident System started. Initially it was difficult for us to get used to the new system, but upon seeing that the chief residents develop their skills of management, education, and mentorship after the completion of those terms, the system became established.

## Study abroad in Canada

**Figure 4:**
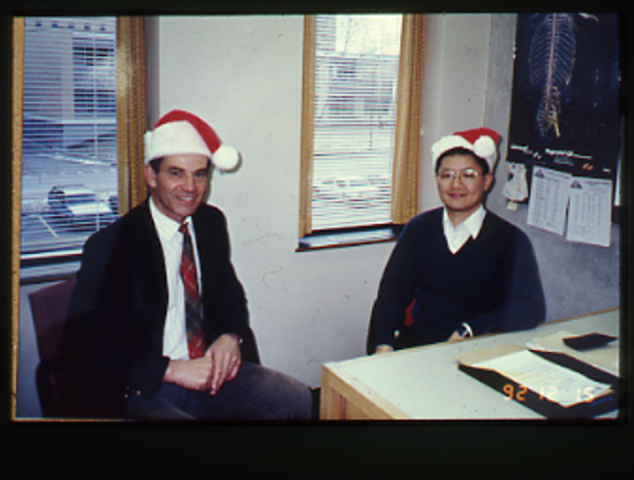
Professor Robert G. Lee and me in his office at the University of Calgary during Christmas season

**Figure 5:**
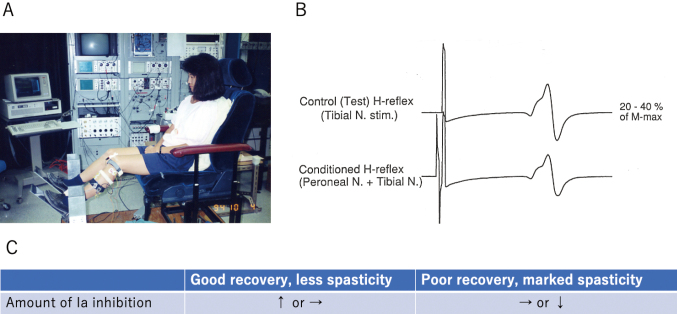
A. Experimental setting to record H-reflex: the posterior tibial nerve is stimulated and H-reflexes are recorded from the soleus muscle. B. Upper wave: the test H-reflex Lower wave: peroneal nerve conditioned H-reflex is slightly depressed. The decrement indicates reciprocal Ia inhibition from ankle flexors to extensors. C. Amount of reciprocal Ia inhibition in the legs with good recovery or poor recovery

I had a chance to study abroad at the University of Calgary, Alberta, Canada from October 1992 to December 1994 with the recommendation by Professor Mizuno. My mentor was Professor Robert Lee, a neurologist in the Department of Clinical Neurosciences (Figure 4). Because both of us were neurologists, we got along very well. I studied reciprocal inhibition in hemiplegia and paraplegia using the H-reflex technique. Figure 5 shows the experimental setting in the laboratory and H-reflex recordings. Because Professor Reisaku Tanaka went to Calgary several years before and he established the laboratory to study the spasticity of patients with spinal cord injury, it was not difficult to start my experiments. My first project was analyzing the reciprocal inhibition in hemiplegic patients following a stroke^2)^. We found that the amount of Ia inhibition from peroneal nerve afferents to soleus motoneurones was increased in patients who were showing good recovery with mild spasticity; however, it did not change or even sometimes diminished in patients who made a poor recovery with marked extensor spasticity (Figure 5-C). The increased amount of Ia inhibition during recovery may be a mechanism to compensate for the loss of descending motor commands. The second project was on the search for the reciprocal Ia inhibition in patients with asymmetric spinal spasticity^3)^. The Ia inhibition to the soleus motoneurones in the legs with good recovery and less spasticity was pronounced, but the Ia inhibition in more spastic legs was small or absent (Figure 5-C). Again, the increased amount of Ia inhibition may be a plastic change to compensate for the less descending motor commands^3)^.

Aside from my research, I enjoyed attending the Clinical Neurosciences Grand Rounds every Friday morning. The rounds took place mainly at Foothills Hospital and they started with two case presentations on either neurology, neurosurgery, or pediatric neurology, followed by the guest speaker’s presentation. One day, a girl showing paraparesis that worsened in the afternoon but improved after sleep was presented in the clinical round. I pointed out that she might have Segawa’s disease (hereditary progressive dystonia with marked diurnal fluctuation)^4)^, which was discovered by the late Professor Masaya Segawa and is well known in Japan. I was very proud to make a diagnosis of this Japanese-discovered disease. Just before returning to Japan, I presented the results of my two-year research work that I performed in Calgary at the Grand Round. At that time, my English has improved enough for the scientific presentation and question-and-answer sessions.

**Figure 6:**
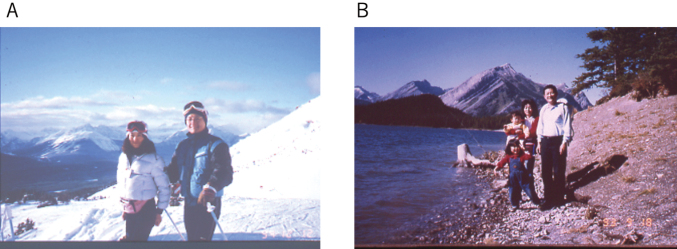
A. Skiing at Lake Louise resort in Canadian Rockies with my wife B. Hiking in the Canadian Rockies with my family

My family and I enjoyed life in Calgary very much. We went skiing in the Canadian Rockies in the winter and hiking in the summer (Figure 6). The people in the neighborhood were kind to us, perhaps because most of them were immigrants. A description of my life in Calgary was partly published in “Sagai”, the alumni magazine of the Faculty of Medicine, Juntendo University, in January 1994.

## Returning to Japan

After returning to Japan, I worked in the Department of Neurology, Juntendo Urayasu Hospital for two years. I continued performing electrophysiological studies of patients with the encouragement from the director, Professor Shigeki Tanaka. We published two articles on “Familial cortical tremor with epilepsy”^5, 6)^, that is now widely known as benign adult familial myoclonic epilepsy (BAFME). I thank Professor Yoichiro Kamiyama for allowing us to use a room that belongs to the Department of Anesthesiology for electrophysiological studies. Since we did not have any rooms specialized for such studies, it was very helpful.

In 1997, I returned to Hongo-Ochanomizu Campus, and served as the senior neurologist to manage human resources of the Department of Neurology as direction of the medical office under the guidance of Professor Mizuno. It was hard work, but I learned how to manage things in the department, and this experience later helped me when I became the director of the Department of Neurology, Juntendo Izu-Nagaoka Hospital and the dean of the Faculty of Health Science and Nursing. I therefore thank Professor Mizuno very much for this appointment. Together with administrative works, I continued trying to elucidate the pathophysiology of movement disorders using electrophysiological methods^7-10)^.

## From Hongo to Izu-Nagaoka

**Figure 7:**
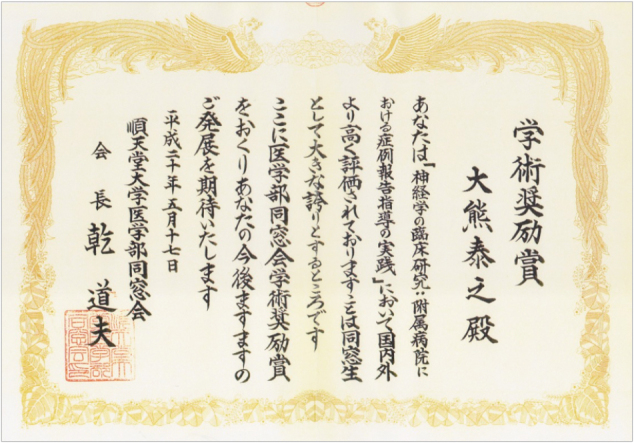
Certificate for Alumni Scientific Award received in 2008

**Figure 8:**
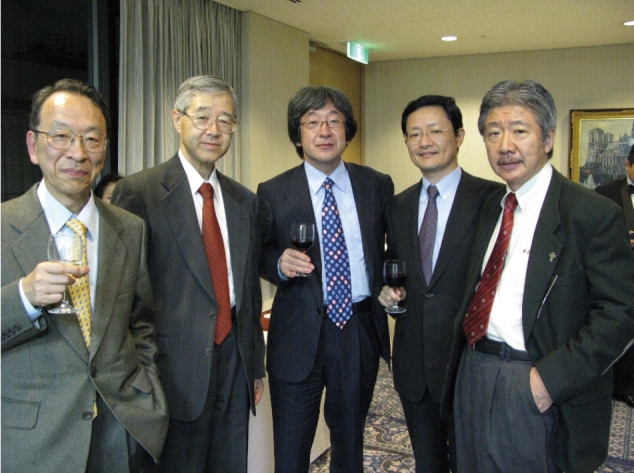
Professor Nobutaka Hattori (center) and associates at the Department of Neurology Alumni Association party in 2008. From left to right, Professor Hisamasa Imai, Professor Masanori Nagaoka, Professor Nobutaka Hattori, the author (Yasuyuki Okuma), and Professor Shigeki Tanaka

In 2000, I was asked by Professor Mizuno to work in Juntendo University Izu-Nagaoka Hospital as the director of the Department of Neurology. The hospital is very active, and I saw many patients suitable for the education of young neurologists. I instructed them to present these cases at the Kanto regional meetings of the Japanese Society for Neurology and to write and submit articles to international academic journals. The first case report was on Neuro-Sweet disease^11)^ presented by Dr. Kazuyuki Noda, the present director of our department. Since then, more than 30 case reports have been published in international journals during my time as the director until 2017^12-44)^. The number of neurologists in our department increased from four to six; thus, we were able to work more actively. Owing to these achievements and faculty development, I received the Alumni Scientific Award in 2008, which was a great honor for me (Figure 7). Professor Nobutaka Hattori (Figure 8), who was the successor of Professor Mizuno, strongly recommended me for the award; thereafter, I was promoted to be a professor of neurology at Juntendo University Shizuoka Hospital in 2009.

As Izu-Nagaoka Hospital, later renamed Juntendo University Shizuoka Hospital, was the core hospital in the eastern part of Shizuoka Prefecture, I aimed to promote the medical care of patients with intractable neurological diseases such as Parkinson’s disease and to educate patients. In cooperation with the Shizuoka branch of the Japanese Parkinson’s Disease Association (JPDA-Shizuoka) and health care centers, we held annual lecture meetings and consultation events for the patients and their family members in Mishima City, Numazu City, and Shimoda City. I also submitted educational articles to the bulletin of JPDA-Shizuoka. As for exercise for the parkinsonian patients, I started to play table tennis with the patients, which I found to be one of the most suitable sports for Parkinson’s disease.

As for research on Parkinson’s disease, I conducted many clinical studies with colleagues of Kanto Parkinson’s Disease Study Group (KPDSG). KPDSG was established in 2004 by Professor Hattori (Juntendo University) and Professor Koichi Hirata (Dokkyo Medical University) to study clinical problems and unmet needs in Parkinson’s disease. The topics included sleep problems, fatigue, postural abnormalities, and body weight loss in Parkinson’s disease. Most research findings have been published in prestigious international journals^45-51)^.

**Figure 9:**
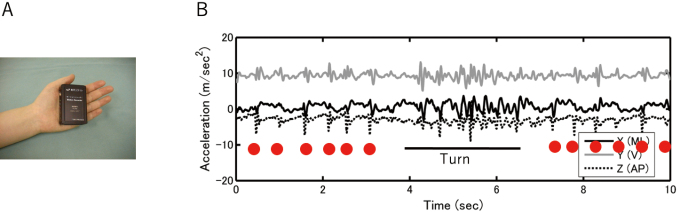
A. 3D accelerometer B. Acceleration recordings during walking and turning in a patient with advanced-stage Parkinson's disease. Red circles show the step rhythm. During turning, high-frequency oscillations corresponding to the freezing of gait were observed.

**Figure 10:**
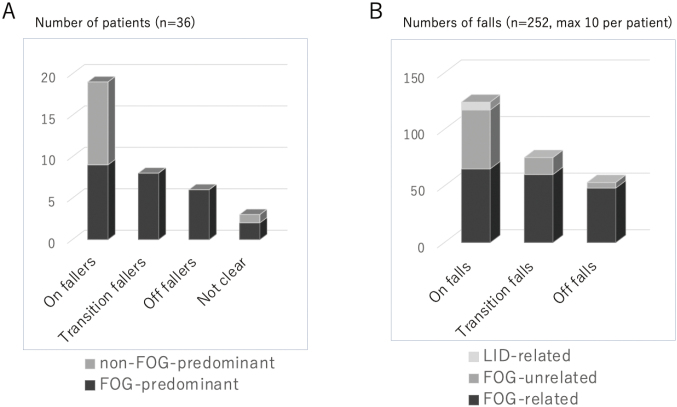
Results of prospective study of falls in Parkinson's disease A. Number of patients for each faller category. Slightly more than half patients (19 patients) are categorized as On-faller. For the Transition-state fallers and Off-fallers, the main cause of falls was freezing of gait. B. Number of falls for each fall category. Only three falls were attributed to severe dyskinesia. The tendency was as the same as Figure 10-A.

Since I was particularly interested in gait disturbance in Parkinson’s disease, I collected data from patients with freezing of gait (FOG) and falls after arriving at Izu-Nagaoka Hospital. I applied neurophysiological methods such as the use of a 3D accelerometer. We found that high-frequency oscillation of accelerations appeared during freezing, and the “freezing index” increased according to freezing episode. We also calculated the body angle so that we could detect falls^52)^. Figure 9 shows the 3D accelerometer and the acceleration with walking steps and freezing episodes occurring while turning in a patient with advanced-stage Parkinson’s disease. In addition to the accelerometric study, I studied the relationships among falls, motor fluctuation, and FOG using a questionnaire. We found that slightly more than half of the patients predominantly fell in the ON-state (patients’ movement is relatively good) among 36 patients^53)^ (Figure 10-A). This may be explained by the fact that the patients’ mobility partially improves but postural instability is generally not adequately improved even at the ON-state in advanced-stage Parkinson’s disease. Only six patients predominantly fell at the OFF- state (patients’ mobility is poor), and the main cause was FOG. These results reflected the fact that our patients were mostly in advanced stages of Parkinson’s disease^53)^. Statistical analyses revealed that a higher number of FOG-related falls occurred in the OFF-state than in the transition and ON- states. In contrast, a higher number of FOG-unrelated falls happened in the ON-state than in the transition and OFF-states (Figure 10-B).

**Figure 11:**
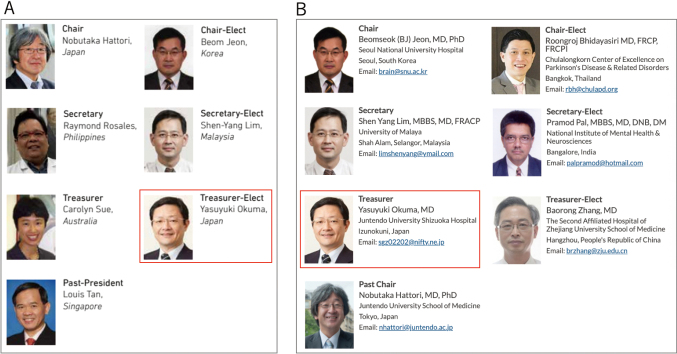
A. Officers of the Asian and Oceanian Section of the International Parkinson and Movement Disorders Society (MDS-AOS) from 2015 to 2017. I served as Treasurer-Elect. Note that Professor Hattori was the Chairman of MDS-AOS. B. Officers of MDS-AOS from 2017 to 2019. I served as Treasurer.

Through these research studies, I became well known as a specialist of gait in Parkinson’s disease among Asian countries. I was then selected as Treasurer-Elect (2015-2017) and Treasurer (2017- 2019) of the Asian and Oceanian Section of the International Parkinson and Movement Disorders Society (MDS-AOS), which was a great privilege for me. Figure 11 shows the members of the section during my terms. Please note that Professor Hattori was the Chairman of the MDS-AOS leaders. I thank Professor Hattori and Professor Mitsutoshi Yamamoto of Takamatsu City for recommending me for an officer of MDS-AOS. It was a precious occasion to work with leaders of neurology in Asian countries.

## Dean of Faculty of Health Science and Nursing

**Figure 12:**
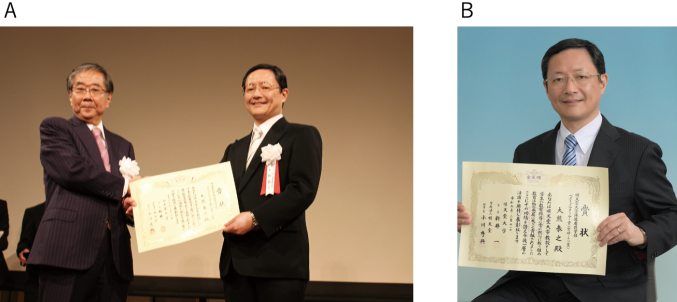
A. Best Professor Prize awarded by CEO Hideoki Ogawa in the thank-you party after the graduation ceremony in March 2018. B. Best Professor Prize in March 2021.

**Figure 13:**
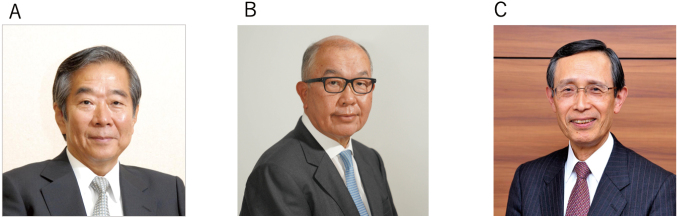
Executive officers of Juntendo University A. CEO, Hideoki Ogawa B. President, Hajime Arai C. Executive Advisor to the President, Eiki Kominami

I was appointed as the dean of the Faculty of Health Science and Nursing in April 2017. This faculty was established in 2010 in Mishima City to produce nurses working in the Juntendo university hospitals. The founding dean was Professor Keiko Inatomi, who was followed by Professor Takao Okada; both previously worked as deans of the Faculty of Health Care and Nursing in the Urayasu Campus. In contrast to those former deans, I had no experience in nursing education. What I could do was only to brush up my lectures to attract nursing students’ interest. Fortunately, since I had many videos of my patients, I used them frequently in my lecture slides, which made my lectures interesting. As a result, I received the Best Professor Award in March 2018 (Figure 12-A). I received the Best Professor Award again in 2021, feeling very proud that I was selected by students, 100% of whom passed the National Examination for Nurses despite the COVID-19 pandemic (Figure 12-B). During my term as the dean, the level of nursing students gradually improved. This was accomplished by recruiting excellent faculty members and faculty development to provide better education, recruiting good students through PR activities, and administering appropriate entrance examinations. Eventually, I was able to complete my term as the dean with the exceptional support and guidance of CEO Hideoki Ogawa, President Hajime Arai, and Executive Advisor to President Eiki Kominami, and I heartfully thank these leaders of Juntendo University (Figure 13). I am very pleased to see the increase in admission capacity and the construction of the new education building in the Mishima Campus, which will be completed in 2024.

**Figure 14:**
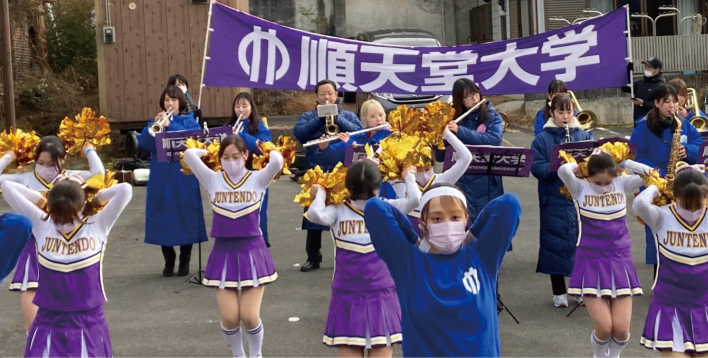
Cheering near the goal of Hakone Ekiden on Jan 2, 2023 at lakeside Ashinoko. The author is the third one from the left in the brass band team

Finally, maybe this is not so important, but I would like to mention Hakone Ekiden, which is one of Juntendo University’s popular sporting events. As I used to play the trumpet in junior and senior high schools, I joined the cheering squad in 2020. Juntendo cheering squad, initially organized by Professor Okada, is quite unique in that most of the members are nursing students. We were allowed to perform cheering for the first time in three years in January 2023 after the long COVID-19 pandemic (Figure 14). Cheering along the roadside gave us much excitement, and we were so moved that our Juntendo Ekiden team won fifth place in the 2023 race.

## Funding

No funding was received.

## Author contributions

YO wrote the whole manuscript, read and approved the final manuscript.

## Conflicts of interest statement

The author declares that there are no conflicts of interest.
